# High resolution 3D mapping of grain kinematics during high temperature sequestration of SO_2_ from flue gas by carbonate aggregates

**DOI:** 10.1038/s41598-020-58216-y

**Published:** 2020-02-10

**Authors:** Mohammad Saadatfar, Frank Brink, Shane Latham, Penelope King, Jill Middleton, Ulrike Troitzsch, Michael Turner, Richard W. Henley

**Affiliations:** 1 Department of Applied Mathematics, Research School of Physics, The Australian National University, Canberra, ACT 2601 Australia; 2 Centre for Advanced Microscopy, The Australian National University, Canberra, ACT 2601 Australia; 3 Research School of Earth Sciences, The Australian National University, Canberra, ACT 2601 Australia

**Keywords:** Chemistry, Energy science and technology

## Abstract

Sulphur dioxide (SO_2_) is removed from flue gases prior to discharge into the atmosphere by high temperature sulphation reactions with the mineral calcite (CaCO_3_) in the form of calcite aggregates such as limestone. The efficiency of this industrial-scale process is constrained by the self-inhibiting growth of anhydrite (CaSO_4_) along calcite grain boundaries. Using very high resolution X-ray μCT and Scanning Electron Microscopy we show, for the first time, how the sulphation reaction is initiated by the anisotropic thermal expansion of calcite grains to produce high inter-grain permeability. In turn fast gas-solid reaction occurs to produce a network of porous anhydrite layers between grains. Individual calcite grains are then free to rotate and translate with respect to each other as the sulphation reaction proceeds. Grain translations of up to 24 μm and rotations of up to 0.64 degrees have been tracked in samples of a highly compacted calcite aggregate (Carrara Marble) across up to 600,000 grains through heating and cooling cycles during exposure to SO_2_ gas flow at temperatures from 600 to 750 °C at one atmosphere. Such grain kinematics help to maintain gas phase permeability in the solid reactant and mitigate the inhibitory growth of porous anhydrite on grain boundaries.

## Introduction

SO_2_ released into the atmosphere by many large-scale industrial processes has the potential to produce acid rain and destroy ozone. Despite increasing regulation of emissions worldwide, about 40 kt of SO_2_ is still released annually into the atmosphere by flue gases from power stations, smelters and sources related to oil and gas production^1^. This suggests that both improved compliance and sequestration efficiencies are required in order to reduce atmospheric discharge of this toxic gas.

The SO_2_ content of flue gases from fossil fuel-fired power stations^2^ may reach about 6000 mg/Nm^3^ with the balance dominated by CO_2_. Currently desulfurization is mostly conducted by wet scrubbing using slurries of calcite aggregate materials, such as limestone or marble^2^, but a proportion is undertaken by the simpler, but less efficient, dry process of reacting high temperature flue gas with a spray of these same raw materials. For this direct-sulphation reaction involving SO_2_ as a gaseous component of the flue gas mixture, the reaction may be written as a disproportionation,

1a $${{\rm{CaCO}}}_{3}+1.5{{\rm{SO}}}_{2}\to {{\rm{CaSO}}}_{4}+{{\rm{CO}}}_{2}+0.25{{\rm{S}}}_{2}$$

This reaction proceeds by chemisorption involving one or more SO_2_ molecules, possibly as dimers, onto the calcite surface^3^ and release of carbon from the lattice. In the industrial process, oxygen is added to minimise the reduced sulphur component of the product gas mixture so that the overall process may, ignoring intermediate gas species, be written,

1b $${{\rm{CaCO}}}_{3}+{{\rm{SO}}}_{2}+0.5{{\rm{O}}}_{2}\to {{\rm{CaSO}}}_{4}+{{\rm{CO}}}_{2}$$

**Figure 1:**
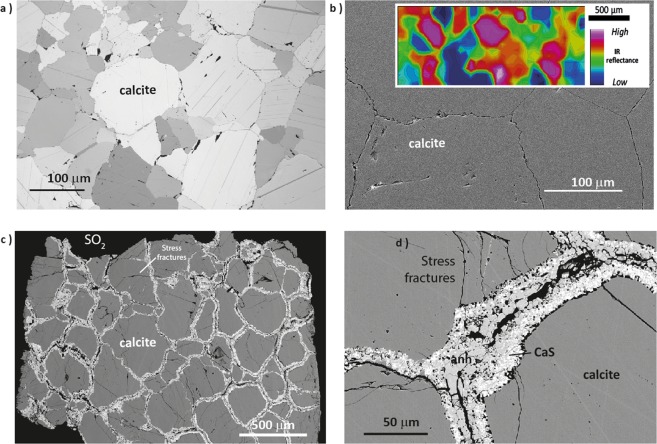
Grain packing and sutured interfaces in unreacted Carrara Marble (**a**) Polarized reflected light, (**b**) Field Emission-Secondary Electron Microscopy (FE-SEM) image. The inset is a Fourier Transform infrared (FTIR) spectral map of the Carrara marble that shows the reflectance intensity of the integrated area under the carbonate stretching band (centred at ~1480 cm^−1^ and measured above a baseline, ~1610–1180 cm^−1^). The colours on the map reveal calcite grains with different crystallographic orientations and sutured grain boundaries with slightly lower topography. (**c**,**d**) Show the intergranular growth of porous anhydrite and minor CaS after 15 days exposure to SO_2_ at 600 °C at 1 atm pressure.

In fixed and fluidized bed^4^ applications, the solid reactant particles are several hundred microns in diameter and are comprised of calcite grains with complex interlocked grain boundaries (Fig. 1a,b). The rate of SO_2_ capture, and consequent process efficiency, is therefore related to progressive changes in the total surface area of the calcite grains that are exposed to reactive gas flow. As the process proceeds this exposure and therefore the effective reaction rate are progressively decreased due to the development of product anhydrite on the ‘shrinking cores’ of calcite grains, so that the yield of anhydrite decreases with time^5–8^. Here we track, at the sub-micron scale, these coupled, rate-limiting chemical and physical processes using a combination of high temperature gas-solid reaction experiments, high resolution micro-computed X-ray tomography (X-ray μCT), Field Emission-Scanning Electron Microscopy (FE-SEM) and X-ray Diffraction (XRD). This approach has enabled us to document, for the first time, the dynamics of individual grain movements (rotation and translation) in a reacting material as a contributing process in maintaining both inter-grain cohesion due to grain boundary interlocking and material permeability. The experimental study provided here is therefore arguably the most detailed record of this, or similar industrial processes involving granular aggregates, and through it, opportunities for increased process efficiencies may be defined.

**Figure 2:**
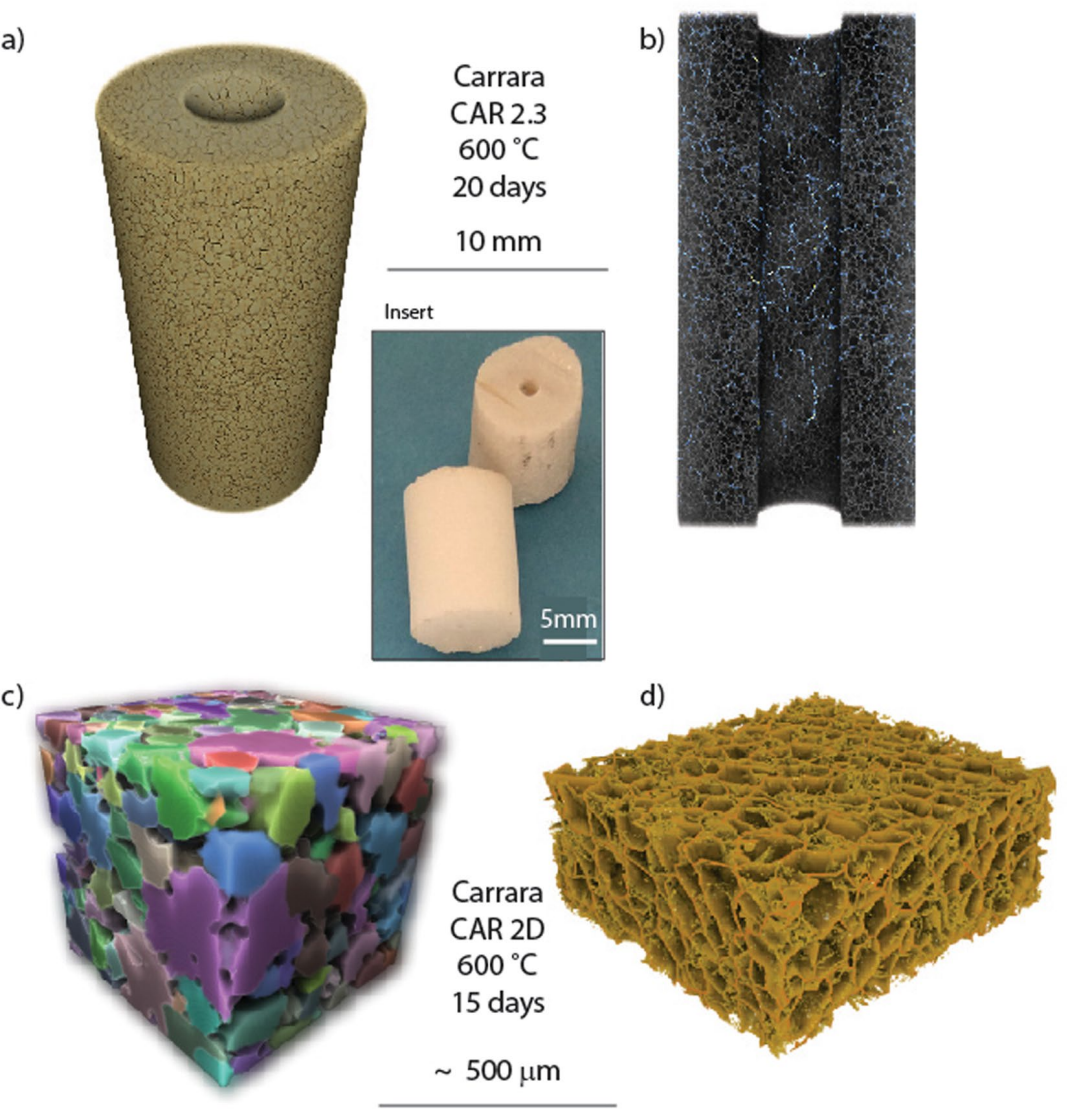
X-ray CT imaging of reacted Carrara marble. (**a**) Definition of grain boundaries through SO_2_ etching, (**b**) internal structure of reacted cylinder showing porous anhydrite (grey) and void space (blue) defining percolation channels, (**c**) X-ray CT image of individual grain packing and (**d**) intergranular porous anhydrite. The inset shows the Carrara Marble hollow cylinders used in these experiments.

We conducted a series of simple experiments to enable tracking of anhydrite product development through repeat exposures of hollow marble cylinders (10 mm external diameter, 4 mm internal diameter, 10–15 mm height – see inset Fig. 2), to a flow of pure SO_2_ at 20 sccm (standard cm^3^ per minute) in a standard vertical one-atmosphere gas-mixing furnace at 600 and 750 °C (Experimental Method and Data Analysis). Reconnaissance experiments at 900 °C are also reported here. X-ray μCT scans were obtained before and after each heating cooling cycle and these data provided 3D mapping of calcite, anhydrite and porosity distributions (tomograms) in the reacted material, grain sizes and the relative movement of calcite grains with respect to one another for each cycle. This provided us with the opportunity, as described below, to track reaction progress across a number of heating and cooling cycles. In a second series of experiments, 5–6 mm thick discs were cut from equivalent cylinders of Carrara Marble. A set of these were hung on a platinum wire in the SO_2_ gas stream in the furnace and then removed one at a time through a sequence of time intervals providing reaction progress data as a function of time at constant temperature. XRD patterns were obtained for each disc and analysed for proportions of anhydrite, remaining calcite and intermediate products.

The reactant material used industrially for SO_2_ sequestration is limestone, a rock material composed primarily of the mineral calcite (CaCO_3_). For the experiments reported here, Carrara Marble was used as a proxy for the range of feedstock limestone used industrially, in view of its purity, highly uniform fabric and relatively even grain size. These are the qualities that make it the choice for classical sculpture such as Michelangelo’s David. The Carrara Bianco Marble that was used in our experiments has very low porosity (<0.004)^9^ and is comprised of closely packed, disoriented calcite crystals with sutured irregular margins (Fig. 1a,b). Its original fabric is a consequence of dynamic recrystallization of high purity limestone at elevated pressure and temperature^10^ such that the material broadly conforms to an intergranular geometry obeying Plateau’s Law of free energy minimisation^11^. The discs and cylinders of Carrara Marble used in these experiments were all cut with the same orientations from the same 75 mm square, 25 mm thick block of Carrara Bianco to minimize small variations in grain size distribution and intergrain tortuosity between samples.

## Results

### Hollow cylinder experiments

Figure 1c and d show the growth of porous intergranular anhydrite (and minor CaS) after 15 days exposure of a cylinder of Carrara Marble to SO_2_ at 600 °C. Only rarely does this anhydrite growth penetrate stress fractures (Fig. 1c) and this suggests, as discussed below, rapid coherent growth of the porous product along the dominant gas percolation channels.

The reaction of calcite aggregate materials to produce intergranular, porous anhydrite introduces X-ray phase contrast (Fig. 2a) enabling individual calcite grains to be identified and tracked through successive stages of reaction using X-ray μCT data. In contrast to standard methods of characterising grain aggregates, such data also provide 3D grain shape, dimension and orientation data over very large grain populations (40,000 to 600,000 in our sub-samples). In essence the gas-solid reaction acts as an etching process through the material that enables discretisation of reactant and product. Phase contrast also allows the recognition of the development of a spongiform intergranular network of porous anhydrite in the product material (Fig. 2b,d) clearly defining the maintenance of a high percolation structure through the reacting material. High resolution FESEM imaging of polished cross sections of the original and successively reacted marble provides detail of the structure of the crystalline anhydrite intergranular product (Fig. 1b–d and distribution of porosity) after one 600 °C heating and cooling cycle.

**Figure 3:**
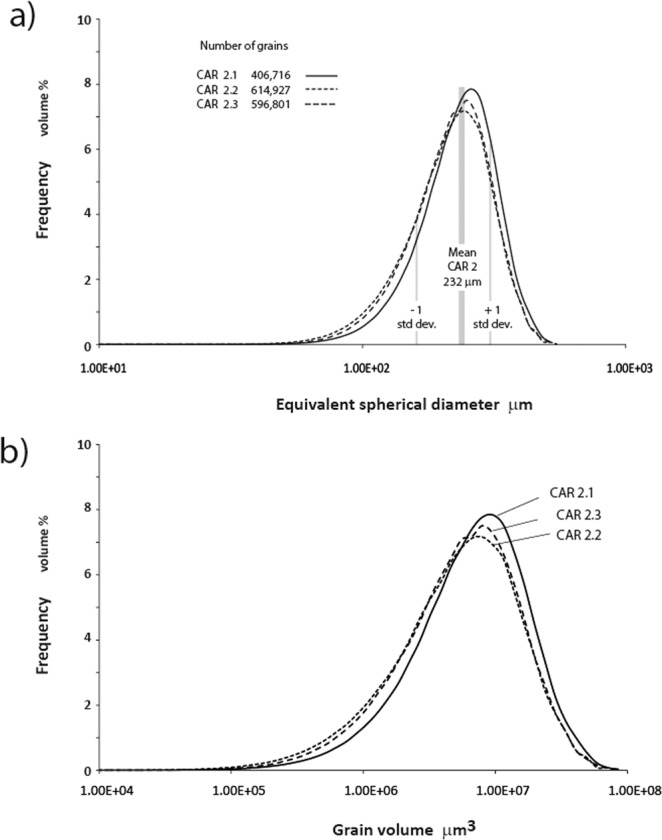
Grain size variations as equivalent spherical volumes and their diameters based on analysis of X-ray μCT data for the exposed Carrara Marble cylinders 2.1 to 2.3 as described in the text.

An additional benefit of the use of high resolution X-ray μCT scanning was new insight into the fabric of such a culturally significant material as Carrara Marble^12^. Grain scale parameterization of Carrara Marble has previously been confined to two-dimensional grain diameter measurements, digital or manual tracing of grain boundaries and statistical analysis using polished or thin sections^13,14^. Grain diameters have commonly been reported as Equivalent Circular Diameters (ECDs), but necessarily, through steric effects^14^, such analysis underestimates actual grain diameters. Making a small correction for intergranular porous anhydrite (12.4 microns) in the reacted material, the X-ray μCT data reported here provide the range of Equivalent *Spherical* Diameters (ESD’s) for the longest cylinder exposure at 600 °C with a mean of 232 microns determined across 596, 801 grains (Fig. 3). Our Carrara Marble may differ from that used for previous published determinations using other samples of Carrara Marble in which median ECD values, determined from grain diameters in 2D sections are 137 μm with standard deviation 74.2 μm^14^ over 220 grains. The data also show a primary fabric of changing grain size which we confirmed with optical imaging at a scale of several cm but which is not evident at the smaller scale of standard polished or thin sections.

**Figure 4:**
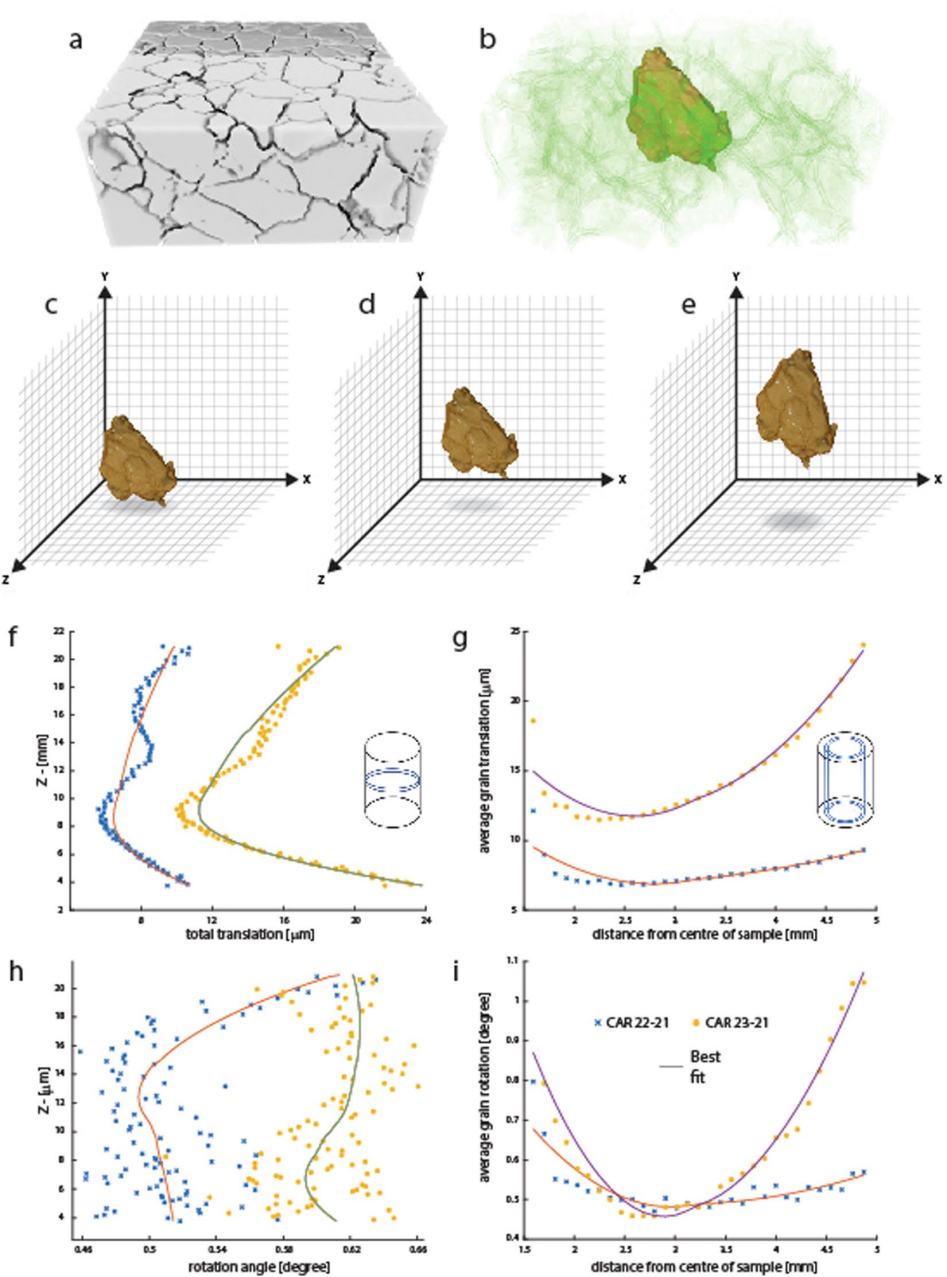
Derivation of grain kinematics from X-ray CT data. (**a**) Sub-sample of a tomogram of a reacted Carrara Marble cylinder with definition of grain boundaries, (**b**) selection of illustrative grain from the centre of the pack, (**c**) to (**e**) illustrate the kinematics of this grain across a reaction cycle with respect to translation in x,y and z and rotation. (**f**) to (**i**) summarised grain translation and rotation data averaged through ~20,000 grain subsets within disk (f&h) and cylindrical (g&i) averaging windows from the entire 596,801 grain population for runs CAR2.2 (yellow) and CAR 2.3 (blue) relative to their distribution in CAR 2.1. These data are shown as average translations in volumes defined as transverse discs and shells as depicted as insets in each figure. Solid lines are best fit curves through these data.

In our experiments, the pervasive development of porous intergranular anhydrite (CaSO_4_) along grain boundaries by reaction with SO_2_ (Fig. 1) releases individual calcite grains within the original grain ensemble as it adjusts to changing distribution of internal stresses during the heating-reaction cycles. Having access to each and every grain’s 3D digital geometry over sample populations of several hundred thousand, we have used a combination of shape factors and tensors of moment of inertia to digitally register individual grains throughout the successive tomograms. These grains can be tracked in the 3D images of the first and the two subsequent runs of the same sample so that the spatial transformation of each grain can be mapped. Figure 3a shows the shape of a single grain of calcite from within a sub-sample of reacted marble CAR 2.3. Mappable translations and rotations are represented within Fig. 4c–e, and quantified in Fig. 4f–i for all grains within the tomogram.

Inhomogeneous regions of high and low (non-affine) displacement are clearly visible at the top and bottom parts of the sample.

Each successive 3D data set may be segmented digitally into non-overlapping discs and shells (Fig. 4f,g) to enable spatial differentiation of grain kinematics. The size of both the non-overlapping disks and cylindrical shells are variable so that these data are reduced to a constant number of grains in these sub-volumes to allow statistical comparison. This provides discrete displacements and rotations for each grain with respect to the tomogram reference system. Figure 4f, for example, shows the average grain displacement along the length of the sample measured across a sequence of contiguous non-overlapping disks. Similarly Fig. 4g shows the average grain displacement measured in cylindrical shells expanding radially from the centre of the sample outwards. Based on these analyses, grains show up to 24 microns of displacement (translation) in both the z (along the length of the sample) and radial (x-y) directions. These values are consistent with the intergrain spacing observed in the FESEM image of a polished slice from this reacted sample. Figures 1d and 4i shows the average grain rotation computed in a similar fashion. Figure 3h shows a z-y slice of grain rotations in the two successive experiments from the initially exposed CAR 21 to the second and third exposures (2.2 and 2.3). The relative errors of each grain motion are negligible due to extremely high precision in measuring grain centroid, radius and surface area (see Experimental Method and Data Analysis for details).

We suggest that, as the samples are unconfined, the irregular grain shapes and crystal orientations of the marble, together with the complex network of inter-connected grain boundaries (Figs. 1 and 2), result in inhomogenous and non-affine grain kinematics during each reaction-heating cycle. Thermal expansion and contraction, coupled with intergranular growth of porous anhydrite, are the principal mechanisms that drive the grain translation and rotation so that primary irregularities in grain shape (see Fig. 3) as well as the disorientation of grain crystallinity (Fig. 1, inset) translate into the temporal non-affine grain kinematics that we have tracked here through segmentation of X-ray CT data across the experimental series.

Due to heating, inter-particle stress develops stress fracturing (Fig. 1d) and increased intergranular permeability that together maximise the exposure of grain surfaces to reactive gas flow. The coefficients of thermal expansion of calcite are +31.78 × 10^−6^ K^−1^ parallel to the c-axis and −3.69 × 10^−6^ K^−1^ at right angles to the crystal c axis^15^ so that even small differences in crystal orientation result in regions of grain boundary extension and compression as well as some grain fracturing as observed in FESEM imaging of reacted material (Fig. 1d). Differential expansion has previously been recognized as the cause of thermal cracking and development of residual strains^16–18^ in the context of degradation of marble surfaces that are exposed to diurnal solar heat cycles up to 130 °C. Here we are able to observe that fracturing occurs before the initiation and growth of the porous intergranular anhydrite matrix (Fig. 1d). This suggests that the initial stages of reaction are fast due to the opening of intergranular porosity by heterogeneous thermal expansion and grain kinematics. This exposes fresh ‘rough’ calcite grain surfaces to gas flow without inhibition by anhydrite product growth. Only some stress fractures become filled with anhydrite which suggests rapid sealing of fractures by porous layer growth (Fig. 1d) as is further discussed below.

### Disc series experiments

**Figure 5:**
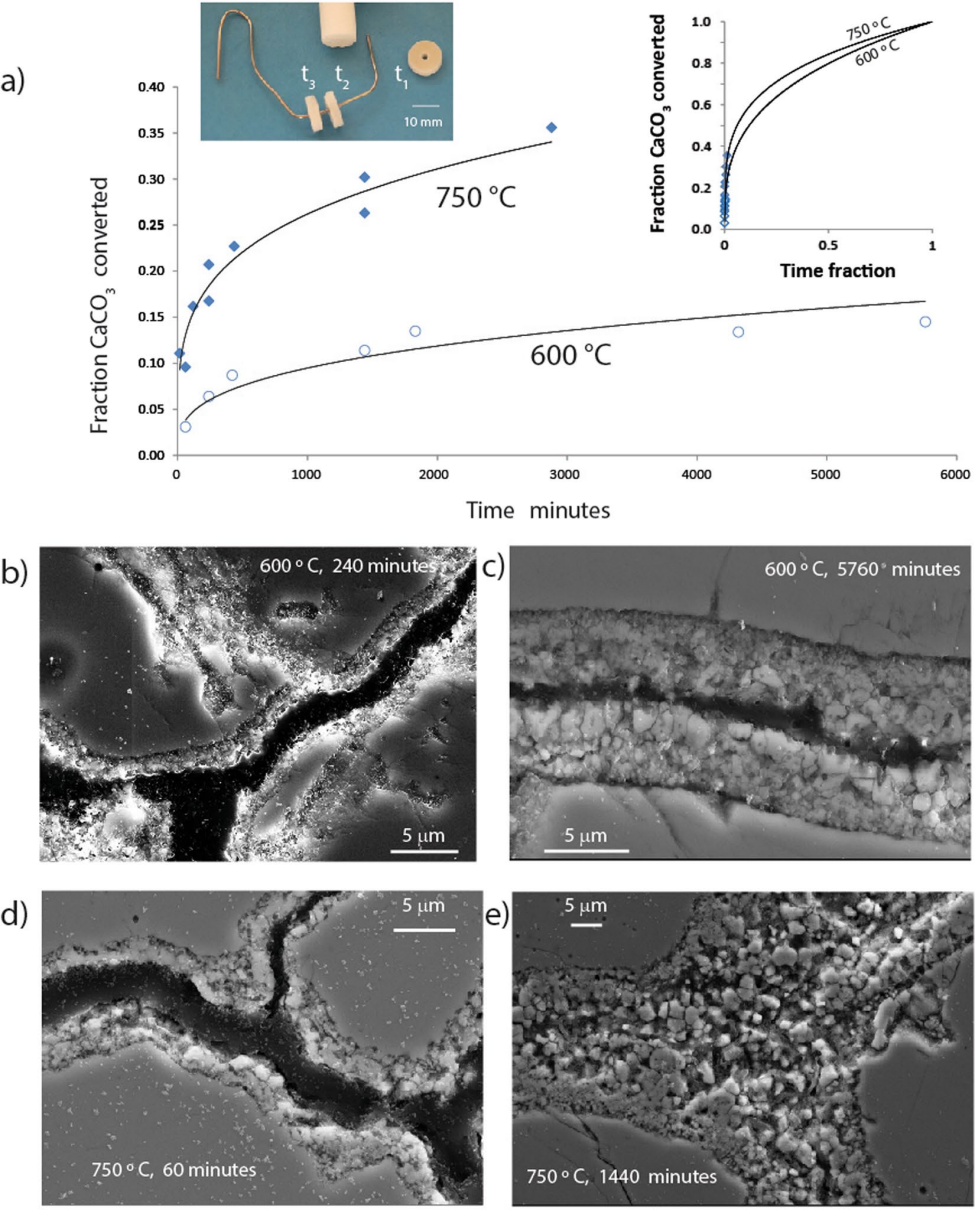
(**a**,**b**) Effective reaction rates determined by experiment on a series of Carrara Marble discs as described in the text. (**b**) to (**e**) show stages of porous anhydrite product development between grains over successive time intervals. The insert shows the experimental set-up with a number of discs cut from a Carrara Marble cylinder and suspended on a bent Pt-wire. Discs are removed from the high temperature furnace gas flow with forceps after times t_1_, t_2_, t_3_, etc.

The separate series of experiments using annular discs of marble provided detail of the progressive growth of the porous intergranular anhydrite and effective reaction rate data (Fig. 5b–e) during exposure to SO_2_ at 600 and 750 °C. These confirmed very high initial reaction rates that decrease rapidly (Fig. 5a) in accord with the general chemical dynamics of shrinking core-expanding rim gas-solid interactions^19–22^. Yield data, expressed as the fraction of initial calcite remaining after each run (Fig. 5a) were obtained from quantitative XRD analysis with estimated standard errors ≤0.3 wt % for the simple mineral component mixture of pure calcite, anhydrite and minor CaS (oldhamite) (see Experimental Method and Data Analysis). This approach assumes similarity of the grain size distribution and intergrain porosity and tortuosity between each disc but as the discs were cut sequentially from the same cylinder of Carrara Marble this is a reasonable assumption.

A time series of calcite conversion rates was undertaken at 900 °C. Almost complete conversion was achieved resulting in a very friable porous product material. In the absence of excess oxygen, CaS reached 10.5 wt % in these experiments compared to a maximum of 1.5 wt % at 750 °C and zero at 600 °C. Up to 2.8 wt % CaO was also present in the 900 °C reconnaissance experiments, suggesting that the principal step in the process at this temperature was thermal decarbonation of the calcite.

## Discussion

The multimodal approach reported here provides new insight to the chemical processes involved in direct sulphation methods of SO_2_ removal from flue gas that complement previous studies^3^. Application of high resolution X-ray μCT is particularly effective in tracking the evolution and distribution of porosity in anhydrite product layers and the behaviour of calcite grains during heating and cooling cycles during sulphation.

The chemical dynamics of calcite particle conversion to anhydrite for a shrinking core-expanding rim gas-solid interaction may be expressed as,

2 $${t}_{(X)}={r}_{k}{[1-X)]}^{1/3}+{r}_{D}^{3}[1-{(1-X)}^{2/3}-\frac{2}{3}X]$$

where *t*_*(X*)_ is the time for conversion of the fraction, *X*, of the starting material, and *r*_*k*_ and *r*_*D*_ are respectively kinetic constants for the reaction rate and layer-diffusion resistance. We suggest that, as the marble discs used in the experiments are unconfined, the irregular grain shape (see Fig. 1) and random crystal orientation of the marble (Fig. 1) results in inhomogenous expansion as the sample heats from room to furnace temperature in each reaction cycle. Grain separation by expansion at time t = 0, exposes calcite surfaces to flow of SO_2_ and initiates anhydrite formation through a percolation network of flow channels (Fig. 2b) so that *r*_*D*_ is minimised and yield is determined by the reaction rate of the thermochemically-favorable sulphation reaction. Anhydrite layer growth then progressively lowers calcite exposure as well as limiting the flux of SO_2_ to reaction surfaces and the counter diffusive flux of product CO_2_ and S_2_. These data therefore provide an explanation of the apparent product layer diffusion inhibition data observed by thermogravimetric experiments^23^. The X-ray μCT data also provided total surface area to volume ratios for the reacted marble disc samples that translate into specific surface area estimates of 1.5–1.8 × 10^7^ μm^2^ per gram calcite. Based on these values the yield data (Fig. 4e) provide estimates of initial effective anhydrite yield rates, for pure SO_2_ reactions, of 10^−14^ moles per m^2^ at 600 to 750 °C. In industrial operations, the effective rate is then a function of the mole fraction (2.8 × 10^−5^ to 2.1 × 10^−4^) of SO_2_ in the CO_2_ -dominated flue gas.

**Figure 6:**
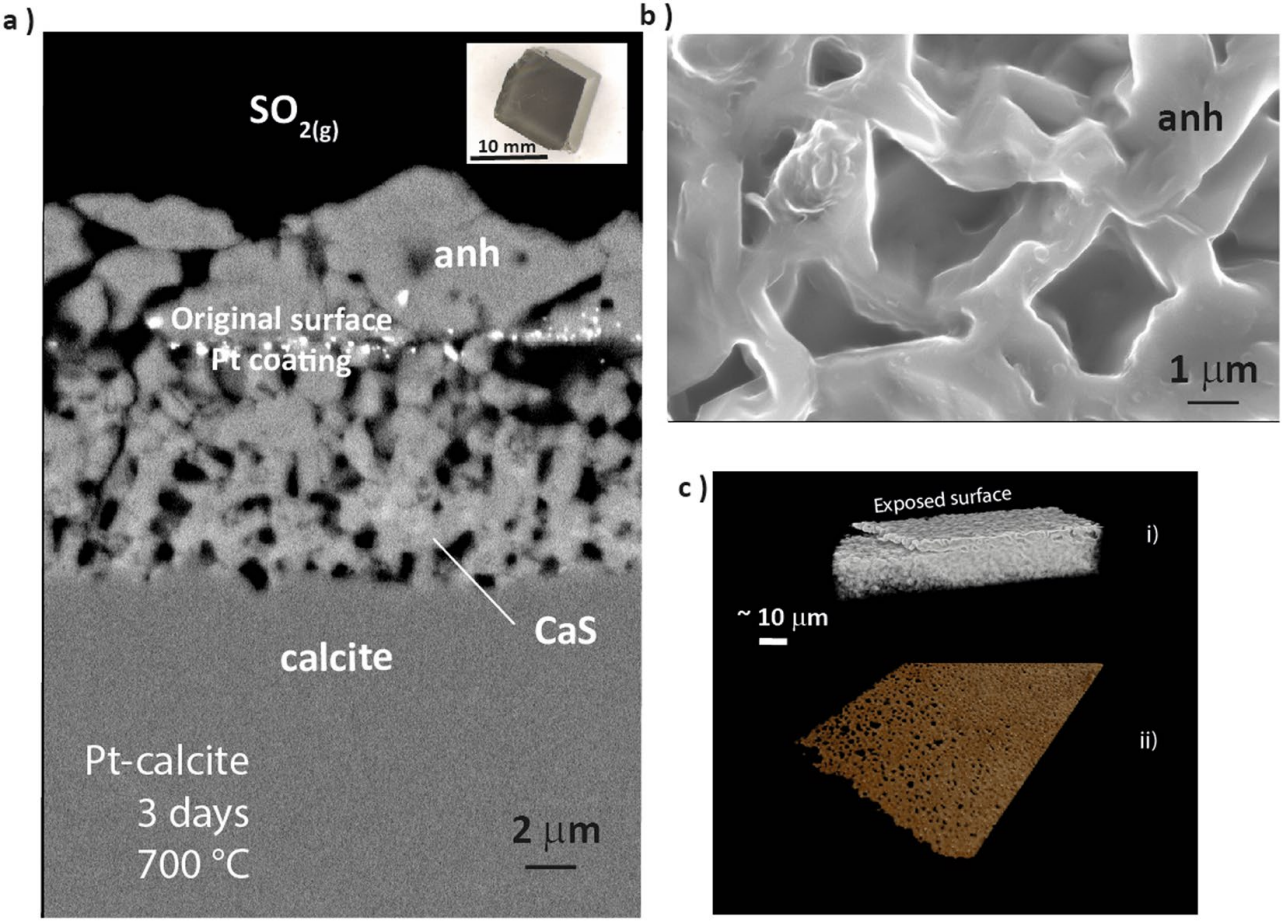
(**a**) Transverse section of the growth of porous anhydrite and minor CaS by sulphation of pure calcite (inset: the original rhomb of Iceland Spar used in the experiment) with respect to the primary crystal face marked by a platinum coating. (**c**) Shows the interlocking crystallite growth in the surface layer above the original surface and (**d**) provides detail of the layer structure and distribution of porosity using sub-micron resolution X-ray CT.

Intergranular growth of porous anhydrite is a major factor in determining reaction yields as a function of time. For Carrara marble, high resolution FE-SEM imaging of polished sections of the disc sample sequence of reacted samples provided detail of the mineralogy and structure of the porous intergranular sulphate-rich cement in relation to unreacted calcite(Figs. 1b and 4b–e). Anhydrite grain growth is coarser and more coherent along the axis of the intergranular porous anhydrite (Fig. 4). This is also shown by layer growth on a platinum-coated cleavage rhomb of pure calcite (Fig. 6). The primary calcite surface was Pt-coated and immediately above the coating a coherent surface layer of anhydrite formed consisting of a mesh of large (~1 μm) crystallites (Fig. 6b,c) with interlocking 1 to 2 μm lozenge-shaped pores (Fig. 1d). The surface of this lattice is decorated with anhydrite apophyses (Fig. 6b) indicative of continuing crystal growth or transformation. Below the Pt-coated original surface, the calcite-anhydrite reaction interface in all cases is cuspate and curved with respect to void space and comprises a jumble of disoriented anhydrite crystal fragments which appear to form by necking-off of anhydrite epiphytes grown from the calcite surface. These cross-sections show that the reaction proceeds rapidly by epitaxial nucleation of anhydrite crystallites directly onto the calcite surface as is described elsewhere for similar, but oxygenated, experiments^24^. Epiphyte crystallization probably occurs by nanoparticle growth^25,26^.

Our interpretation is that layer structure evolves as the reaction front migrates inward from the primary surface so that the the coarse grained lattice may be considered to be formed by coalescence of earlier epiphytes. This layer progressively restricts the flux of reactant and product gases to and from the retreating calcite surface and may be the major cause of the observed progressively decreasing effective reaction rate and the increasing dominance of the diffusion term as layer growth proceeds (Fig. 5). Gas flux decrease at the calcite reaction surface is also suggested by the spatial relationship between CaS, CaSO_4_ and the reaction interface (Figs. 1 and 5). The formation of CaS suggests relative starvation of the reaction front to SO_2_ as a consequence of layer cementation, progressive porosity reduction and dilution of SO_2_ at the reaction front by product gases. CaS is an intermediate product of the reaction through,

3a $$4{{\rm{CaCO}}}_{3}+4{{\rm{SO}}}_{2}=3{{\rm{CaSO}}}_{4}+{\rm{CaS}}+4{{\rm{CO}}}_{2}$$

3b $${\rm{CaS}}+2{{\rm{SO}}}_{2}={{\rm{CaSO}}}_{4}+{{\rm{S}}}_{2}$$

These partial equations combine to the synoptic form of Eq. (1) for sulphation in the absence of excess oxygen.

$${{\rm{CaCO}}}_{3}+1.5{{\rm{SO}}}_{2}\to {{\rm{CaSO}}}_{4}+{{\rm{CO}}}_{2}+0.25{{\rm{S}}}_{2}$$

CaS has much lower abundance than CaSO_4_ in the yield experiments (see Experimental Methods and Data Analysis). The decarbonation rate of calcite increases with temperature so that the observed minor proportion of CaO below ~700 °C progressively increases and becomes dominant over CaS at 900 °C (see Experimental Methods and Data Analysis). Trace amounts of hannebachite (CaSO_3_.0.5H_2_O) occurred only in some of the experiments which we interpret as the hydration product of CaS due to exposure to moist air during sample extraction from the furnace, and provided the smell of H_2_S as exposed samples were withdrawn at each stage of the experimental sequence. These and other published data^3,24,27^ suggest that CaSO_3_ is not an intermediate product in the sulphation of limestone^28^. Similar layer growth is likely to occur during sulphation of oxygenated flue gas but CaS and other reduced sulphide species are necessarily suppressed.

Above the original crystal face in the calcite rhomb experiment, and distal to the reaction front, the anhydrite growth layer comprises larger grains and a complex ‘chicken wire’ fabric. It is evident that calcium migration has occurred through, and likely from, the lower layer. It is possible that some Ca migration may occur through pore space as CaS given the above observations. Another possibility is that Ca diffusion to the outer growth surface occurs as a consequence of lattice defects in the rapidly grown epiphytes at the calcite reaction surface.

In summary, the application of a multimodal approach to tracking the progress of high temperature direct sulphation reactions using calcite aggregates such as Carrara Marble has provided new data on the dynamics of shrinking core, expanding rim gas-solid reactions involving particle aggregates. The use here of quantitative X-ray μCT analysis in particular has also provided new perspectives on material properties during heterogeneous gas reactions through quantifying the temporal and spatial changes in the distribution of individual grains and their shapes and volumes over populations of several thousand grains. These kinematics have not previously been quantified and are therefore not accommodated in process modelling.

From the data developed here on self-inhibition due to growth of porous anhydrite on all grain boundaries, it becomes immediately apparent why the spray dry scrubbing method of SO_2_ sequestration from flue gas is relatively inefficient compared to wet scrubbing methods^1^. Optimisation of the direct process might be achieved, as shown by our 900 °C experiments (Table 1), by maintenance of higher temperatures in the gas-particle stream^29^. Decarbonation at this temperature achieved almost complete conversion of the calcite aggregate through conversion to CaO which then has a higher reaction rate with SO_2_. However increasing temperature in a reducing gas stream may limit sulphur capture^30^. In some cases it has been suggested that use of solid CaO (lime) or partially calcined limestone^31^ may be a more efficient method (Indirect sulphation) for high temperature SO_2_ sequestration but of course this may involve some energy as well as economic penalties. Flue gas SO_2_ sequestration operations are necessarily site specific (and much more varied in the higher sulphur context of smelting operations where conversion to sulphuric acid is often economically viable) but the recognition of the self-inhibiting reaction mechanism may suggest possibilities for recycling of reacted aggregate via crushing to expose fresh calcite to flue gas. Indeed, in fluidized beds, this may well be accomodated by autogenous milling, a possibility that might also be investigated using the multimodal approach described here.

**Table 1:** XRD mixture analyses for Carrara Marble disc reactions with SO_2_ at 600, 750 and 900 °C.

Experiment	Temperature	Run time	CaCO3		CaSO4		(Ca-Mg)S		CaO		MgO	
Number	°C	Minutes	wt%	esd	wt%	esd	wt%	esd	wt%	esd	wt%	esd
CAR_F1	600	240	93.6	0.3	6.4	0.3	0		0		0	
CAR_F2	600	420	91.3	0.3	8.7	0.3	0		0		0	
CAR_F3	600	1440	88.6	0.3	11.4	0.3	0		0		0	
CAR_F4	600	1830	86.5	0.2	13.5	0.2	0		0		0	
CAR_F5	600	2880	86.8	0.3	13.2	0.3	0		0		0	
CAR_F6	600	4320	86.6	0.2	13.4	0.2	0		0		0	
CAR_F7	600	5760	85.5	0.3	14.5	0.3	0		0		0	
CAR_C1	750	1440	79.5	0.2	19.0	0.2	1.5	0.1	0		0	
CAR_C2	750	2880	74.8	0.2	23.9	0.1	1.3	0.1	0		0	
CAR_C3	750	5760	71.5	0.1	27.2	0.1	1.3	0.1	0		0	
CAR_D1	750	60	90.4	0.2	9.2	0.1	0.4	0.1	0		0	
CAR_D2	750	120	83.8	0.2	15.7	0.2	0.5	0.1	0		0	
CAR_D3	750	240	83.2	0.2	16.2	0.2	0.6	0.1	0		0	
CAR_D4	750	435	77.3	0.1	22.1	0.1	0.6	0.1	0		0	
CAR_D5	750	1440	69.8	0.1	29.0	0.1	1.2	0.1	0		0	
CAR_D6	750	2880	64.4	0.1	34.5	0.1	1.1	0.1	0		0	
CAR_E1	900	120	0.3	0.1	88.6	0.1	10.4	0.1	0.1	0.1	0.5	0.2
CAR_E2	900	267	1.6	0.1	87.2	0.2	8.8	0.1	0.9	0.1	0.3	0.1
CAR_E3	900	399	0.3	0.1	84.3	0.2	9.6	0.1	2.3	0.1	0.5	0.2
CAR_E4	900	1440	0.4	0.1	82.8	0.2	9.6	0.1	2.8	0.1	0.6	0.2
CAR_E5	900	1860	1.7	0.1	87.0	0.2	10.5	0.1	0.2	0.1	0.4	0.1
CAR_E6	900	2940	0.1	0.1	89.2	0.2	10.2	0.1	0.1	0.1	0.3	0.1

Note the almost complete conversion of calcite to anhydrite at 900 °C with CaS occurring to about 10 wt% in the product mixture due to the absence of added oxygen, and that CaO was an intermediate product due to decarbonation of the calcite. esd = estimated standard deviation.

## Methods

In order to provide a high-purity starting material of relatively uniform grain size, we selected Carrara Bianco Marble (White). This material was used in experimental determination of the influence of deformation on porosity and permeability in calcite aggregates by^32^ and is similar to that described by Leiss and Weiss^16^ for their analysis of fabric anisotropy. The calcite crystal experiment was conducted using transparent rhombs of Iceland Spar. The inset to Fig. 1b is an Infra-red (IR) map of a polished section of Carrara Marble that shows the variation of reflectance intensity of the area under the 880–890 cm^−1^ band (ν_2_ out-of-plane-bend for CO_3_^2−^) and shows these as a colour spectrum where each colour band indicates a different crystal orientation. Specular reflectance spectra were collected on a Bruker Tensor 27 spectrometer attached to a Hyperion microscope at the Research School of Earth Sciences at ANU. The instrument uses a Globar source, XT-KBr beam-splitter and MCT-A detector and spectra were collected from 4000–650 cm^−1^, with a 4 cm^−1^ resolution and 200 scans. The IR beam was apertured to a ~35 × 35 µm square area and overlapping spectra collected (x-step = 25 µm and y-step = 32 µm). The intensity of the integrated area under the carbonate stretching band was contoured with the OPUS 7.0 software.

Sulphation experiments were conducted using a vertical muffle tube furnace (Gero GmbH) heated by MoSi_2_ elements. Temperature was controlled using a Eurotherm PID controller reading a sheathed Type B thermocouple located approximately in the hot spot, external to the muffle tube. The offset between the control thermocouple temperature and sample temperature was calibrated by measuring temperature profiles inside the muffle tube. Gas flow was maintained by a Tylan General F2800 mass flow controller at a rate of 20 sccm.

Samples were prepared as 10 mm diameter cylinders or discs of Carrara Marble each with 4 mm central holes to improve gas flow and to provide suspension in the furnace. Samples were suspended in the hot spot of the furnace by a Pt-Rh wire coil attached to an alumina rod. The sample was introduced into the furnace at 600 °C, and the temperature was ramped at 6 °C/min to experimental temperature, at which point the SO_2_ flow was started. The SO_2_ gas used in all experiments was 99.9% minimum purity and supplied by BOC (Australia). At the end of the experiment, the sample was dropped to the colder bottom of the muffle tube. The furnace was then purged with Ar gas, and the sample was extracted. Reaction yield data were obtained using a series of 10 mm diameter discs of Carrara marble each with a central hole suspended together in the gas stream, with successive removal disc by disc and gravimetric determination of weight gain or loss combined with quantitative X-ray determination of the changes in composition as a function of run time.

3D mineral distribution data were obtained by Xray µ-CT using a combination of helical scanning, image registration and segmentation for quantitative analysis as described by^33^. These data were obtained on the custom-built facility in the Department of Applied Mathematics at the Research School of Physics at the Australian National University. This instrument is capable of imaging materials with a wide variety of densities and sizes, from 5 cm cores of rock, imaged at 20 μm resolution, down to samples less than 5 mm across, imaged with a voxel size of less than 2 μm. The scan data quantitatively analyzed using in-house developed Mango and Drishti visualization software^34^.

The Carrara Marble tomograms contain up to 600,000 grains depending on the size of the sample and image resolution. The calcite grains are digitally separated using a set of algorithms developed at the Australian National University^35,36^. The average grain diameter is about 230 micrometres (see Fig. 3a) i.e. ~53 voxels across. Given the voxel size of 4.37 micrometres, therefore an average calcite grain is represented by a cluster of approximately (4/3)*π*(53/2)^3^ ≈ 78,000 voxels and the grain’s surface corresponds to a cluster of 4*π*(53/2)^2^ ≈ 8,800 voxels. A grain centre is the geometric centroid of the 78,000 voxel coordinates that belong to the grain, that is, the grain centre is an average quantity computed from these large clusters of voxels that represent each grain. As a consequence of the large voxel representation of a grain’s volume, the resolution on the grain centre determination is extremely high, i.e. greater than 10^−4^  μm. The precision (typical error) on the centroid determination is related to the segmentation of the voxels that cover the surface of a calcite grain. The segmentation process using our in-house software^27^ is very robust and it ensures that the precision of our measurements is comparable to our resolution within a factor of order unity.

For the disc series of experiments, backscattered electron and field emission-secondary electron images (Fig. 5b–e) were obtained using a Zeiss Ultraplus field emission-scanning electron microscope (FESEM). Operating conditions were 15 kV and 1 nA. Powder XRD was carried out to identify phases in the Carrara marble discs before and after SO_2_-treatment. The original unreacted Carrara marble sample was milled to < 10 µm and the powder suspended on a side-packed sample holder. It was analyzed with a Siemens D5005 Bragg Brentano diffractometer (reflection geometry) using *CuKα* radiation, a graphite monochromator and a scintillation detector. The scan range was 4 to 84° 2θ at a step width of 0.02° 2θ and collection time of 4 seconds per step. Quantitative XRD analysis was undertaken using Siroquant V3 software within which errors for derived weight percentages of each solid reactant and product are estimated through a least-squares variance-covariance matrix approach. Estimated standard deviations (esd’s) are provided below in Table 1.

Effective reaction rate calculations were conducted using these and gravimetric data for the samples before and after each experimental run. Gravimetric errors were negligible at 6 × 10^−3^%. The compound error for derived anhydrite yields are up to 0.8% where this error is at its maximum for short run time experiments with low reaction yield relative to longer run times. The variation of anhydrite yield per heating-cooling cycle may be determined as the ratio of new mole % anhydrite relative to the percentage of remaining calcite in the preceding disc, with all data normalised back to a starting point of 1 mole of pure calcite. This assumes that the grain size variations and consequent grain surface areas are similar for each Carrara Marble disc and this assumption is supported by the grain size parameter data in Fig. 3. Cumulative conversion rates (expressed as the ‘fraction of CaCO_3_ converted’ in Fig. 5a) are calculated directly from the XRD analyses. Yield data discussed here have been derived by reference to the actual weight of each disc before and after each cycle, but in a routine industrial application XRD data normalised step by step back to an initial 1 mole of calcite (or total Ca) would provide satisfactory data.

## Data Availability

The datasets generated during and/or analysed during the current study are available from the corresponding author on reasonable request.
